# Inter-method reliability of the modified Rankin Scale in patients with subarachnoid hemorrhage

**DOI:** 10.1007/s00415-021-10880-4

**Published:** 2021-11-08

**Authors:** E. Nobels-Janssen, E. N. Postma, I. L. Abma, J. M. C. van Dijk, R. Haeren, H. Schenck, W. A. Moojen, M. H. den Hertog, D. Nanda, A. R. E. Potgieser, B. A. Coert, W. I. M. Verhagen, R. H. M. A. Bartels, P. J. van der Wees, D. Verbaan, H. D. Boogaarts

**Affiliations:** 1 Department of Neurology, Canisius Wilhelmina Hospital, Nijmegen, The Netherlands; 2 Department of Neurosurgery, Radboud University Medical Center, PO Box 9101, Nijmegen, 6500 HB The Netherlands; 3 Amsterdam UMC, Department of Neurosurgery, Amsterdam Neuroscience, University of Amsterdam, Amsterdam, The Netherlands; 4 IQ Healthcare, Radboud University Medical Center, Radboud Institute of Health Sciences, Nijmegen, The Netherlands; 5 Department of Neurosurgery, University Medical Center Groningen, Groningen, The Netherlands; 6 Department of Neurosurgery, Maastricht University Medical Center, Maastricht, The Netherlands; 7 Department of Neurosurgery, Haaglanden Medical Center, The Hague, The Netherlands; 8 Department of Neurosurgery, Leiden University Medical Center, Leiden, The Netherlands; 9 Department of Neurosurgery, Haga Teaching Hospital, Leiden, The Netherlands; 10 Department of Neurology, Isala Hospital, Zwolle, The Netherlands; 11 Department of Neurosurgery, Isala Hospital, Zwolle, The Netherlands

**Keywords:** Modified Rankin Scale, Reliability, Subarachnoid hemorrhage

## Abstract

**Background and objectives:**

The modified Rankin Scale (mRS) is one of the most frequently used outcome measures in trials in patients with an aneurysmal subarachnoid hemorrhage (aSAH). The assessment method of the mRS is often not clearly described in trials, while the method used might influence the mRS score. The aim of this study is to evaluate the inter-method reliability of different assessment methods of the mRS.

**Methods:**

This is a prospective, randomized, multicenter study with follow-up at 6 weeks and 6 months. Patients aged ≥ 18 years with aSAH were randomized to either a structured interview or a self-assessment of the mRS. Patients were seen by a physician who assigned an mRS score, followed by either the structured interview or the self-assessment. Inter-method reliability was assessed with the quadratic weighted kappa score and percentage of agreement. Assessment of feasibility of the self-assessment was done by a feasibility questionnaire.

**Results:**

The quadratic weighted kappa was 0.60 between the assessment of the physician and structured interview and 0.56 between assessment of the physician and self-assessment. Percentage agreement was, respectively, 50.8 and 19.6%. The assessment of the mRS through a structured interview and by self-assessment resulted in systematically higher mRS scores than the mRS scored by the physician. Self-assessment of the mRS was proven feasible.

**Discussion:**

The mRS scores obtained with different assessment methods differ significantly. The agreement between the scores is low, although the reliability between the assessment methods is good. This should be considered when using the mRS in clinical trials.

**Trial registration:**

www.trialregister.nl; Unique identifier: NL7859.

**Supplementary Information:**

The online version contains supplementary material available at 10.1007/s00415-021-10880-4.

## Introduction

The modified Rankin Scale (mRS) is one of the most frequently used outcome measures in randomized clinical trials in patients with an aneurysmal subarachnoid hemorrhage (aSAH) [1]. The mRS is an ordinal 7-point scale ranging from no residual symptoms (0), to severely disabled (5) or death (6). The mRS measures the constructs: mobility, disability in basic and instrumental activities of daily living, and living arrangements (Table 1) [2]

**Table 1:** The mRS score [2]

Score	Meaning
0	No symptoms
1	No significant disability despite symptoms. Able to carry out all usual duties and activities
2	Slight disability. Unable to carry out all previous activities, but able to look after own affair without assistance
3	Moderate disability. Requiring some help, but able to walk without assistance
4	Moderately severe disability. Unable to walk without assistance and unable to attend to own bodily needs without assistance
5	Severe disability. Bedridden, incontinent and requiring constant nursing care and attention
6	Death

Given the broad use of the mRS as a primary endpoint for aSAH in randomized controlled trials, it is important to assess its reliability. In clinical trials it is often not described how the mRS is assessed, while the method might influence the mRS score [3, 4]. There are several possible assessment methods of the mRS. First, a regular non-structured assessment by a physician or nurse practitioner, in which the mRS is often scored in hindsight after an appointment to the outpatient clinic. Second, a face-to-face structured interview can be used to score the mRS. The structured interview consists of specific questions per mRS score. It has been shown that the inter-rater reliability of the mRS in patients after stroke is better with a structured interview than with conventional scoring, although some studies show conflicting results [3, 5, 6]. Third, a self-assessment by the patient (either online or on paper) is a reported possibility of mRS measurement in stroke [7]. Next to these different structural methods, the assessment can be conducted in person or by telephone. The aim of this study is to evaluate the inter-method reliability between a structured interview or self-assessment of the mRS compared to a regular assessment by a physician.

## Methods

### Study design and participants

This prospective, multicenter, randomized study was registered in the Netherlands Trial Register (NTR number NL7859). Between November 2018 and September 2020, patients were recruited from six hospitals in the Netherlands. Patients were eligible for this study when they had a recent diagnosis (≤ 6 weeks) of aneurysmal subarachnoid hemorrhage and were aged ≥ 18 years. Patients who were non-fluent in Dutch or not able to visit the outpatient clinic for follow-up were excluded. Due to the possibility of introducing bias, it is not possible to evaluate the mRS by structured interview and by self-assessment in the same patient. Therefore, enrolled patients were randomized for either the structured interview group or the self-assessment group. Given the nature of the assessment method, blinding was not possible. Online block randomization was used, with stratification for institutes. Ethical approval was not required for this type of study under Dutch law, and an exemption was obtained by the local Medical Ethics Committee (CMO region Arnhem-Nijmegen, file number 2018-4184). All patients or their representatives gave written informed consent.

### Procedures

Data were collected at two time intervals after the aSAH: approximately after 6 weeks and 6 months in accordance with standard Dutch care after aSAH. Demographic information (age, sex), date of aSAH, World Federation of Neurosurgery Score (WFNS) on admission, modified Fisher score, location of the aneurysm, and date of discharge were obtained from digital medical records. At 6 weeks and 6 months after inclusion, the attending physician assigned the mRS score first to reduce the risk of bias. This was done face-to-face or by telephone. No specific rules for this assessment were set, since the goal was to evaluate the usual standard of care assessment of the mRS. Within 2 weeks after this appointment, patients took part in the structured interview or completed the self-assessment. The Dutch version of the structured mRS interview was used, which was previously translated according to the existing guidelines for translation [8]. All assessors of the structured interview were trained by an online learning module for the assessment of the mRS in advance of the study. In most centers, there was one assessor. Only in the two centers with the most inclusions there were two or three assessors, mostly specialized nurses or physician assistants. The preferred assessment method for the structured interview was a face-to-face interview. When this was not possible, a telephone interview was scheduled. The self-assessment was completed during an outpatient appointment, or was sent to the patient’s home address. When a patient had a missing answer on one or multiple questions of the self-assessment, the mRS was counted as missing when an answer on the missing question would have resulted in a different mRS score. Feasibility of the self-assessment was tested using a feasibility questionnaire with questions concerning time, difficulty, understanding and emotional burden.

### Data analysis

Before the start of the study, a sample size was calculated. The expectation was to be able to see patterns of distribution across mRS scores and to calculate a Cohen’s kappa with 60 patients per group. Using a twenty percent non-compliance percentage to the study protocol, a total of 150 patients had to be included. Analyses were conducted with IBM SPSS version 25. Missing data were deleted pairwise. Causes for missing mRS scores were categorized in: death, withdrawal of consent, organizational issues including rescheduling appointments due to COVID-19 pandemic, no follow-up indicated by physician, no-show by the patient, or missing answers to questions on the self-assessment. Descriptive statistics were used to describe participant characteristics. The main outcome measure is the weighted kappa score (quadratic) between the mRS scored by a physician and the mRS scored by structured interview, and the weighted kappa between the physician score and the self-assessment score. The quadratic weighted kappa score assigns lower weights to greater discrepancies compared to smaller discrepancies. Following standard protocol, a kappa of 0 to 0.2 was considered poor, 0.21 to 0.4 fair, 0.41 to 0.6 moderate, 0.61 to 0.8 good, and 0.81 to 1.0 excellent [9]. The weighted kappa was calculated using http://www.vassarstats.net/kappa.html [10, 11]. Patients that had the structured interview or self-assessment more than 14 days after the assessment of the physician were excluded from the analysis, since it is not guaranteed that a difference in mRS score is caused by a difference in scoring rather than by a change in health status. Percentage agreement was calculated, as well as the specific agreement between different mRS scores [12]. Specific agreement is the observed agreement relative to each rating category individually and can be used for ordinal scales (comparable to positive and negative agreement for binary ratings). This can be calculated by comparing one mRS score versus any of the others, but also by comparing one mRS score with one of the others. The Wilcoxon test for paired groups was used to analyze whether there was a systematic difference between the assessment methods.

## Results

In total, 150 patients were included in this study, of whom 26.2% were male and the mean age was 58.3 years (range 22–83) (Table 2). One of the included patients was in hindsight given a diagnosis of non-aneurysmal subarachnoid hemorrhage and was, therefore, excluded from all analyses. At 6 weeks, there were 135 valid assessments by the physician, of which 77 patients (57.0%) were scored by telephone, 56 patients (42.2%) with a face-to-face assessment and in one patient (0.7%) the mRS score was based on the answers of a proxy. At 6 months, 134 valid assessments by the physician were available, of which 86 (64.2%) telephone assessments, 45 (33.6%) face-to-face assessments, and in 3 patients (2.2%) another assessment method was used, for example a video call. The flowchart in the supplemental material (Online Figure I) shows the missing data and included number of patients in the analyses.

**Table 2:** Patient characteristics

	Total (*n* = 149)	Structured interview (*n* = 75)	Self-assessment (*n* = 74)
Age	58.3^1^ (11.0)	57.3^1^ (10.5)	59.4^1^ (11.6)
Sex			
Male	39 (26.2%)	19 (25.3%)	20 (27.0%)
Female	110 (73.8%)	56 (74.7%)	54 (73.0%)
Center			
RUMC	52 (34.9%)	26 (34.7%)	26 (35.1%)
UMCG	8 (5.4%)	4 (5.3%)	4 (5.4%)
AMC	59 (39.6%)	30 (40%)	29 (39.2%)
HMC	11 (7.4%)	5 (6.7%)	6 (8.1%)
Isala	14 (9.4%)	7 (9.3%)	7 (9.5%)
MUMC	5 (3.4%)	3 (4.0%)	2 (2.7%)
Location of aneurysm			
Anterior circulation	98 (65.8%)	47 (62.7%)	51 (68.9%)
Posterior circulation	46 (30.9%)	25 (33.3%)	21 (28.4%)
Unknown	5 (3.4%)	3 (4.0%)	2 (2.7%)
WFNS grade			
I	75 (50.3%)	43 (57.3%)	32 (43.2%)
II	29 (19.5%)	13 (17.3%)	16 (21.6%)
III	10 (6.7%)	5 (6.7%)	5 (6.8%)
IV	20 (13.4%)	9 (12.0%)	11 (14.9%)
V	15 (10.1%)	5 (6.7%)	10 (13.5%)
Modified Fisher score			
0	1 (0.7%	1 (1.3%)	0
1	13 (8.7%)	6 (8.0%)	7 (9.5%)
2	22 (14.8%)	11 (14.7%)	11 (14.9%)
3	46 (30.9%)	22 (29.3%)	24 (32.4%)
4	63 (42.3%)	31 (41.3%)	32 (43.2%)
Missing	4 (2.7%)	4 (5.3%)	0

*AMC* Amsterdam medical center, *HMC* Haaglanden medical center, *MUMC* Maastricht university medical center, *RUMC* Radboud university medical center, *UMCG* University medical center Groningen, *WFNS* World Federation of Neurosurgical Societies

^1^Mean (standard deviation)

### mRS distribution

**Fig. 1:**
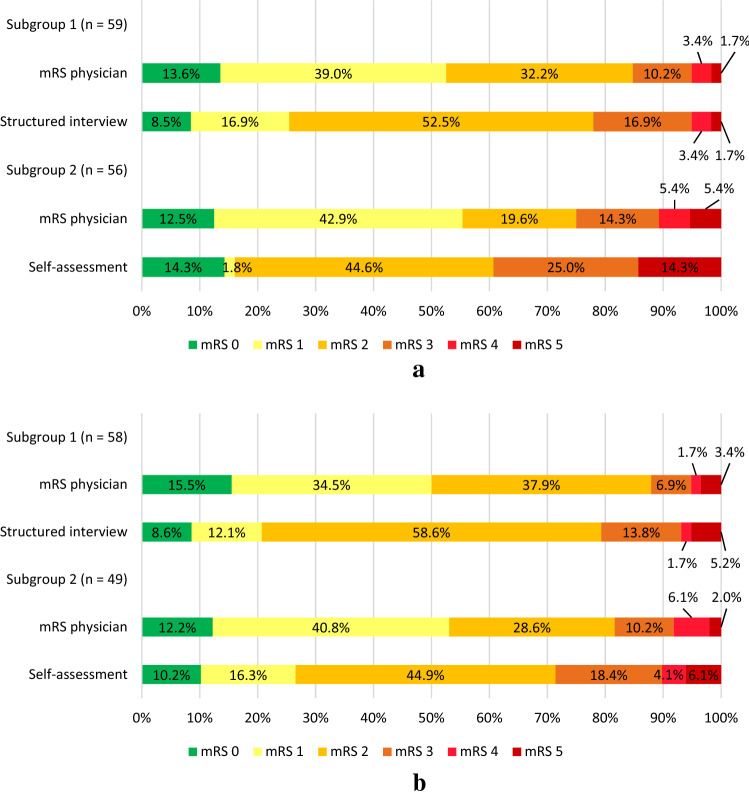
Frequency distribution of mRS scores at 6 weeks (**a**) and 6 months (**b**) assigned by the physician or based on a structured interview or self-assessment

The distribution of the mRS shows a non-normal left-skewed distribution, with a range of scores 0 to 5 and most scores clustering around mRS 2. The mRS scores obtained from a structured interview were systematically higher than the mRS scored by the physician at 6 weeks (median score of 2 vs 1, respectively; *Z* = − 3.0; *p* = 0.002) (Fig. 1a). The same applied for the score obtained from self-assessment compared to the mRS score of the physician at 6 weeks (median score of 2 vs 1; *Z* = − 3.6; *p* < 0.001). At 6 months the systematic differences in scores persisted (structured interview compared to the physician: median mRS score 2 vs 1; *Z* = − 4.1; *p* < 0.001; self-assessment compared to physician: median mRS score 2 vs 1; *Z* = − 2.8; *p* = 0.004).

### Reliability

Perfect agreement between the mRS scored by the physician and the structured interview was present for 50.8% of mRS scores at 6 weeks (Tables 3, 4 and Online Tables I–III). The weighted kappa was 0.60, which is considered a moderate agreement. The percentage agreement between the mRS scored by the physician and a self-assessment at 6 weeks was much lower (19.6%), with a weighted kappa of 0.56 (moderate agreement). The disagreement was greater for some category boundaries than others (Table 4, Online Tables I–III). The specific agreement whether patients scored an mRS 0 or 1 by the physician compared to mRS scores 0 or 1 based on the structured interview at 6 weeks was 56.6%, and the specific agreement between mRS 0–2 scores by the physician and mRS scores 0–2 based on the structured interview was 91.7% (Table 4). It is more difficult to distinguish an mRS score of 1 from an mRS score of 2 (specific agreement 53.3%), than to distinguish an mRS score of 1 from 0 (specific agreement between scores of 88.9%) (Table 4).

**Table 3:** Reliability and agreement parameters for the different assessment methods

	6 weeks			6 months		
	Percentage agreement	Unweighted kappa (95% CI)	Weighted kappa (95% CI)	Percentage agreement	Unweighted kappa (95% CI)	Weighted kappa (95% CI)
mRS physician compared to structured interview	50.8%	0.33 (0.16–0.51)	0.60 (0.17–1.00)	41.4%	0.18 (0.00–0.35)	0.69 (0.56–0.83)
mRS physician compared to self-assessment	19.6%	0.05 (0.00–0.17)	0.56 (0.36–0.77)	42.9%	0.26 (0.08–0.44)	0.59 (0.22–0.95)

*CI* confidence interval, *mRS* modified Rankin Scale

**Table 4:**
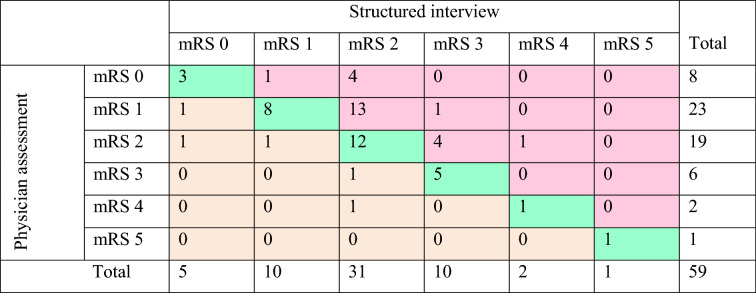
Comparison of mRS scores assigned by the physician and structured interview at 6 weeks

*mRS* modified Rankin Scale

The green shaded boxes show exact agreement between the scores of the physician and structured interview. The red shaded boxes are the mRS scores that were scored higher by structured interview. The orange shaded boxes are the mRS scores that were scored higher by the physician

In 61.3% of the cases, there was a difference of 1, 2 or 3 scores in the mRS score (as shown in Table 4, online Tables I–III and summarized in Table 5). In 79.4% of differences, the mRS score based on the structured interview or self-assessment was higher than the score of the physician. The main reasons for a lower mRS score assigned by the physician compared to a structured interview or self-assessment at 6 weeks and 6 months were because patients indicated that they encountered problems with participation (mRS 2) (57.4%) or the need for assistance with some instrumental activities of daily living (iADL) (mRS 3) (28.7%) (Table 4, Online Tables I–III). Participation problems identified with structured interview or self-assessment were mostly based on problems with return to previous work or difficulties with previous social and leisure activities, and problems with iADL were mainly the need for assistance for doing household chores, looking after money and travelling locally. Notably, in seven cases, the patient indicated needing constant care in their self-assessment, while this was not scored as such by the physician. This happened once in the structured interview group (Online Tables I–III). We have no insight in the main reasons for a higher mRS score assigned by the physician compared to the other assessment methods, since we did not ask the physician for an explanation of the assigned mRS score. For self-assessment and structured interview, an mRS score of 0 was assigned 12 times, with a corresponding physician score of mRS 1 (9 times) or mRS 2 (3 times). When a patient was assigned an mRS score of 1 (by structured interview or self-assessment) the corresponding physician score was 2 (4 times) or 3 (once), for an mRS 2, the physician scored 3 (8 times) or 4 (once), and it occurred once that a patient scored an mRS 3 on self-assessment and the physician scored a 4.

**Table 5:** Frequencies of differences in mRS score specified

	Physician versus structured interview		Physician versus self-assessment		Total
	Six weeks (*n* = 59)	Six months (*n* = 58)	Six weeks (*n* = 56)	Six months (*n* = 49)	
Difference of 1 mRS level	21 (35.6%)	30 (51.7%)	30 (53.6%)	19 (38.8%)	100 (45.0%)
Negative difference	18	25	21	13	77
Positive difference	3	5	9	6	23
Difference of 2 mRS levels	8 (13.6%)	4 (6.9%)	13 (23.2%)	8 (16.3%)	33 (14.9%)
Negative difference	6	4	11	7	28
Positive difference	2	0	2	1	5
Difference of 3 mRS levels	0	0	2 (3.6%)	1 (2.0%)	3 (1.4%)
Negative difference	0	0	2	1	3
Positive difference	0	0	0	0	0
Total	29 (49.2%)	34 (58.6%)	45 (80.4%)	28 (57.1%)	136 (61.3%)
Negative difference	24	29	34	21	108
Positive difference	5	5	11	7	28

*mRS* modified Rankin Scale

Absolute numbers are presented and percentages compared to the total number of valid measurements. In the situation that the physician scored the mRS lower than the mRS based on the structured interview or self-assessment, it is called a negative difference. If the mRS scored by the physician is higher than the mRS based on the structured interview or self-assessment, it is called a positive difference

### Feasibility of mRS self-assessment

The response rate for the mRS self-assessment at 6 weeks and 6 months was very high (resp. 91.9% and 87.8%). The proportion of missing values at 6 weeks and 6 months was resp. 16.2 and 20.0%. Most patients indicated that the self-assessment of the mRS was completely clear (resp. 71.2% at 6 weeks and 80.6% at 6 months). Patients generally found the questions very easy to answer (resp. 75.8 and 74.2%) and the majority of patients indicated that they understood all questions (resp. 72.7 and 90.3%). Most patients did not think it took a long time to answer the questionnaire (resp. 88.9 and 87.1%) and found the assessment not at all emotionally demanding (resp. 75.8 and 82.0%). Patients with missing answers on the self-assessment had comparable scores on the items of the feasibility questionnaire to patients without missing data (< 3% difference per answer category).

## Discussion

This study shows that the reliability between different assessment methods of the mRS is moderate for use in patients with an aSAH. However, the percentage agreement between the different assessment methods is low, especially between the physician score and the self-assessment. The mRS scores obtained with different assessment methods differ significantly, with the physician systematically assigning lower mRS scores compared to both structured interview and self-assessment.

Cohen’s kappa is a coefficient of agreement for categorical outcomes and it incorporates a correction for agreement occurring by chance, which is dependent on the heterogeneity of the sample [13]. The percentage agreement is an absolute measure and is the measure of choice for questions about decision-making in individual patients. In clinical practice in general, assessments are performed to diagnose and monitor individual patients, and therefore differences between scores can have direct implications for the care that individual patients receive. The quadratic weighted kappa is the main outcome measure in this study and shows a moderate reliability between the different assessment methods. Our results illustrate that the weighted kappa is more or less the same between a structured interview and the physician’s assessment and between self- assessment and the physician’s assessment, despite relevant differences in percentage agreement. Since the mRS is not used as an instrument to monitor individual patients, but to assess outcome as a group, it is more important that the reliability of the mRS is high rather than the percentage agreement. However, since the method of assessment is relevant for the outcome of the mRS, the assessment method of the mRS should always be described in the methods section of trials. Furthermore, it should be recognized that the mRS scored by a structured interview or a self-assessment gives structurally higher mRS scores than those scored by a physician. Comparison of scores obtained with different assessment methods is therefore not valid.

The scores on the mRS are frequently dichotomized in clinical trials and dichotomization often occurs using different composite scores, for example cutoff at 0–3 and 4–6 or cutoff at 0–2 and 3–6. In randomized controlled trials in patients with aSAH, different methods of dichotomization are used and some studies even created three new categories or only reported the upper and lower ordinal categories [1]. The non-linear distribution of the mRS and the variability in the interpretation of disabilities, result in a lower specific agreement especially in the mRS scores 1, 2 and 3 [6]. That means that a small difference in this midrange of mRS scores, which can be caused by a low specific agreement, can lead to a shift between these two outcome groups. Since our study shows that the specific agreement around these intermediate grades is relatively poor, use of a structured assessment of the mRS and the use of non-dichotomized data would lead to more accurate results. Recently, in acute stroke trials a shift towards non-dichotomized data and even the use of a utility-weighted mRS has been made [14]. A utility outcome is a representation of the desirability of that specific health outcome to a patient, with a utility of 1 representing excellent health and a score of 0 presenting a health situation equal to death.

Self-assessment of the mRS is feasible and the inter-method reliability compared to the assessment of a physician is moderate. This study shows that at 6 weeks after aSAH the agreement between a structured interview and the physician was higher than the agreement between a self-assessment and the mRS scored by the physician. A head-to-head comparison of a structured interview with self-assessment is necessary to assess the inter-method reliability between these two assessment methods, although previous research in patients with aSAH showed that an online self-assessment has an excellent inter-method reliability compared to a telephonic interview [7]. The reason for higher mRS scores by self-assessment compared to the physician were patient-reported problems with participation or the need for assistance with some iADL tasks. Based on the data of this study we cannot determine which of the assessments is “right”: whether the higher mRS score represents the real situation after the aSAH, or that the difference in scores rather represents the underlying comorbidity, the legal requirements related to the ability to drive or a wrong interpretation of the question by the patient. One way to assess this could be to evaluate with physicians and patients after the assessment why they answered the questions in the way they did. As a next step, a discussion about the provided answers with both physicians and patients could be initiated and an evaluation whether they would adjust their answer based on the input of the other. However, this was beyond the scope of this investigation. An mRS score of 5 is scored more often by self-assessment than by structured interview or by the physician. This score represents a patient who is dependent on continuous care and usually bedridden. Corresponding mRS scores by the physician are mRS 3 or mRS 4. Therefore, we think the difference might be based on an estimation of the severity of physical complaints or on cognitive dysfunction leading to a need for continuous supervision, although we cannot state which score reflects the actual situation. Our experiences during this study revealed that during self-assessment patients who did not yet return to their previous occupation, answer the question about restrictions or problems with work with an answer that indicates no limitations, although not being able to return to work is also a restriction in the ability to work. This misinterpretation illustrates the importance of a thorough cognitive validation study in patients before implementing a self-assessment in clinical studies. Additionally, it is important to realize that patients with aphasia, with decreased insight into their own performance or with other cognitive impairments, might not be able to complete a self-assessment. In these cases, a proxy (e.g. a family member) might be able to answer the questions. How to handle this should be considered before implementing a self-assessment in clinical studies.

The administration of a structured interview of the mRS in large clinical trials can be experienced as time consuming, although it generally does not take more than 5–10 min per person. Therefore, the simplified modified Rankin Scale (smRSq) was developed [15]. It consists of five questions addressing the key functional states assigned to each mRS score and it is usually assessed through a patient interview. In the future, the smRSq might replace the structured interview of the mRS due to the shorter administration time, although currently it is not clear whether the smRSq measures the same construct as the mRS. Correlation with EQ-5D utility score was comparable for smRSq and conventional non-structured mRS scoring [16]. However, in several studies, the distribution of patients over the mRS categories differed between the smRSq and mRS, with more patients scoring mRS 3 compared to conventional scoring [16, 17]. Therefore, more research is necessary to evaluate validity of the smRSq, especially focusing on the question whether the smRSq measures the same construct as the mRS.

To be able to compare and pool results of clinical trials, a core outcome set was defined for outcome measures to implement in studies in patients with aSAH [18]. The mRS was considered a preferred outcome measure and classified as Supplemental—Highly Recommended. Our study underlines the importance of using non-dichotomized mRS data and describing the assessment method. Researchers should be aware that mRS scores obtained with different assessment methods are not necessarily comparable. The risk for introducing bias is especially large in retrospective studies or database studies, in which different assessment methods may have been used. For these reasons, it is advisable to use a structured interview to assess the mRS.

### Limitations

Our study has several limitations. First, based on our results we are not able to state that the reliability of one of the assessment methods is higher than one of the others since no repeated measures were done per assessment method. Additionally, we were not able to state with certainty which assessment method is most valid, i.e. closest to the actual situation. Due to the COVID pandemic, we were forced to make more telephone appointments instead of physical consultations at the outpatient clinic. This may have influenced the results, although previous research showed that there is good agreement between a telephone assessment and a face-to-face assessment of the mRS with a structured interview [8].

Second, the three ways of assessment of the mRS should ideally have been evaluated in the same patient. However, this would introduce a risk of bias, because answers on the structured interview or self-assessment could have influenced the answers on the next assessment. Therefore, we consciously chose to use two randomized groups to avoid this form of bias.

Third, although our study population is an adequate representation of patients that survived the aSAH, a limited number of patients with an mRS of 3, 4 or 5 was included in this study [19, 20]. This may limit the generalizability of the results to severely affected patients with aSAH. However, previous literature shows that the agreement in mRS scores is high for the mRS scores 3–5 [6, 21].

Finally, we set no limitations to the total number of participating raters for either the physician’s scores or the structured interview. We know from previous research that, considering the moderate inter-rater reliability, studies with multiple raters are prone to more variability. Since all raters on the structured interview were trained beforehand this will limit variability. On the other hand, this study design represents the actual situation for most clinical trials at this moment and therefore improves external validity.

## Conclusions

The results of this study show that reliability between different assessment methods of the mRS is moderate, and the mRS scores assessed by a structured interview or self-assessment are systematically higher compared to assessment by a physician in the follow-up of aSAH patients. Therefore, studies using different assessment methods are not comparable. Future research using the mRS as outcome measure should clearly describe the assessment method and preferably determine scores with a structured interview. Dichotomization of the mRS should be avoided. Finally, self-assessment of the mRS is feasible for use in clinical trials.

## Supplementary Information

Below is the link to the electronic supplementary material.

Supplementary file1 (DOCX 1144 KB)

## Data Availability

Study data not provided in this article will be made available by request from any qualified investigator.
