# Supplementary material for: Inter-method reliability of the modified Rankin Scale in patients with subarachnoid hemorrhage
Source: J Neurol. 2021 Nov 8;269(5):2734–42. doi: 10.1007/s00415-021-10880-4 (PMC8572691; doi:10.1007/s00415-021-10880-4)
Supplement: Supplementary file 1 — Supplementary file1 (DOCX 1144 KB) [file 415_2021_10880_MOESM1_ESM.docx]

**SUPPLEMENTAL MATERIAL**

eFigure I: Flowdiagram of included patients and patients lost for analysis

Flow diagram of the randomized patients and patients lost to follow-up or excluded from analysis. Missing data are presented per time interval for all included patients.

eTable I: Comparison of mRS scores assigned by the physician and structured interview at six months.
The green shaded boxes show exact agreement between the scores of the physician and structured interview. The red shaded boxes are the mRS scores that were scored higher by structured interview. The orange shaded boxes are the mRS scores that were scored higher by the physician.

|  | | Structured interview | | | | | | Total |
| --- | --- | --- | --- | --- | --- | --- | --- | --- |
|  |  | mRS 0 | mRS 1 | mRS 2 | mRS 3 | mRS 4 | mRS 5 |  |
| Physician assessment | mRS 0 | 4 | 3 | 2 | 0 | 0 | 0 | 9 |
|  | mRS 1 | 1 | 2 | 15 | 2 | 0 | 0 | 20 |
|  | mRS 2 | 0 | 2 | 15 | 5 | 0 | 0 | 22 |
|  | mRS 3 | 0 | 0 | 2 | 1 | 1 | 0 | 4 |
|  | mRS 4 | 0 | 0 | 0 | 0 | 0 | 1 | 1 |
|  | mRS 5 | 0 | 0 | 0 | 0 | 0 | 2 | 2 |
| Total | | 5 | 7 | 34 | 8 | 1 | 3 | 58 |

Abbreviations: mRS: modified Rankin Scale

eTable II: Comparison of mRS scores assigned by the physician and self-assessment at six weeks.
The green shaded boxes show exact agreement between the scores of the physician and self-assessment. The red shaded boxes are the mRS scores that were scored higher by self-assessment. The orange shaded boxes are the mRS scores that were scored higher by the physician.

|  | | Self-assessment | | | | | | Total |
| --- | --- | --- | --- | --- | --- | --- | --- | --- |
|  |  | mRS 0 | mRS 1 | mRS 2 | mRS 3 | mRS 4 | mRS 5 |  |
| Physician assessment | mRS 0 | 2 | 0 | 3 | 2 | 0 | 0 | 7 |
|  | mRS 1 | 5 | 0 | 14 | 5 | 0 | 0 | 24 |
|  | mRS 2 | 1 | 0 | 5 | 5 | 0 | 0 | 11 |
|  | mRS 3 | 0 | 1 | 3 | 1 | 0 | 3 | 8 |
|  | mRS 4 | 0 | 0 | 0 | 1 | 0 | 2 | 3 |
|  | mRS 5 | 0 | 0 | 0 | 0 | 0 | 3 | 3 |
| Total | | 8 | 1 | 25 | 14 | 0 | 8 | 56 |

Abbreviations: mRS: modified Rankin Scale

eTable III: Comparison of mRS scores assigned by the physician and self-assessment at six months.
The green shaded boxes show exact agreement between the scores of the physician and self-assessment. The red shaded boxes are the mRS scores that were scored higher by self-assessment. The orange shaded boxes are the mRS scores that were scored higher by the physician.

|  | | Self-assessment | | | | | | Total |
| --- | --- | --- | --- | --- | --- | --- | --- | --- |
|  |  | mRS 0 | mRS 1 | mRS 2 | mRS 3 | mRS 4 | mRS 5 |  |
| Physician assessment | mRS 0 | 2 | 1 | 2 | 1 | 0 | 0 | 6 |
|  | mRS 1 | 2 | 5 | 9 | 4 | 0 | 0 | 20 |
|  | mRS 2 | 1 | 2 | 9 | 2 | 0 | 0 | 14 |
|  | mRS 3 | 0 | 0 | 2 | 2 | 0 | 1 | 5 |
|  | mRS 4 | 0 | 0 | 0 | 0 | 2 | 1 | 3 |
|  | mRS 5 | 0 | 0 | 0 | 0 | 0 | 1 | 1 |
| Total | | 5 | 8 | 22 | 9 | 2 | 3 | 49 |

Abbreviations: mRS: modified Rankin Scale
